# Convergent Associative Motor Cortical Plasticity Induced by Conditional Somatosensory and Motor Reaction Afferents

**DOI:** 10.3389/fnhum.2020.576171

**Published:** 2020-10-21

**Authors:** Yi Huang, Jui-Cheng Chen, Chon-Haw Tsai, Ming-Kuei Lu

**Affiliations:** ^1^Graduate Institute of Biomedical Sciences, College of Medicine, China Medical University, Taichung, Taiwan; ^2^Department of Neurology, China Medical University Hospital, Taichung, Taiwan; ^3^Neuroscience and Brain Disease Center, China Medical University, Taichung, Taiwan; ^4^School of Medicine, College of Medicine, China Medical University, Taichung, Taiwan; ^5^Ph.D. Program for Translational Medicine, College of Medicine, China Medical University, Taichung, Taiwan

**Keywords:** intracortical facilitation (ICF), motor evoked potential (MEP), paired associative stimulation (PAS), primary motor cortex (M1), reaction time, short interval intracortical inhibition (SICI)

## Abstract

**Objective**: Associative motor cortical plasticity can be non-invasively induced by paired median nerve electric stimulation and transcranial magnetic stimulation (TMS) of the primary motor cortex (M1). This study investigates whether a simultaneous motor reaction of the other hand advances the associative plasticity in M1.

**Methods**: Twenty-four right-handed subjects received conventional paired associative stimulation (PAS) and PAS with simultaneous motor reaction (PASmr) with at least a 1-week interval. The PASmr protocol additionally included left abductor pollicis brevis muscle movement responding to a digital sound. The motor reaction time was individually measured. The M1 excitability was examined by the motor evoked potential (MEP), short-interval intracortical inhibition (SICI), and intracortical facilitation (ICF) before and after the PAS protocols.

**Results**: The conventional PAS protocol significantly facilitated MEP and suppressed SICI. A negative correlation between the reaction time and the MEP change, and a positive correlation between the reaction time and the ICF change were found in the PASmr protocol. By subgrouping analysis, we further found significant facilitation of MEP and a reduction of ICF in the subjects with fast reaction times but not in those with slow reaction times.

**Conclusion**: Synchronized motor reaction ipsilateral to the stimulated M1 induces associative M1 motor plasticity through the spike-timing dependent principle. MEP and ICF change could represent this kind of plasticity. The current findings provide a novel insight into designing rehabilitation programs concerning motor function.

## Introduction

The motor cortical plasticity contributes to motor learning and appropriate movements (Sanes and Donoghue, 2000; McKay et al., 2002; Dayan and Cohen, 2011; Kida and Mitsushima, 2018; Kroneberg et al., 2018). A specific type of plasticity following Hebb’s theory, or known as the spike-timing dependent principle, has been found existing in the human motor cortex (M1) (Hebb, 1949; Müller-Dahlhaus et al., 2010). Not only has it been observed in the cell level, but also in the systemic and behavioral level (Zhang et al., 1998; Stefan et al., 2000; Bi and Poo, 2001; Dan and Poo, 2004; Cooke and Bliss, 2006; Ziemann et al., 2008). That is, the excitability of M1 can be dependently modulated by repetitive, time-locked pairing stimuli, mostly with one sub-threshold conditioning stimulus and one supra-threshold test stimulus (Müller-Dahlhaus et al., 2010). Paired associative stimulation (PAS) is a non-invasive brain stimulation method commonly applied to modulate M1 excitability (Stefan et al., 2000, 2002). The conventional PAS protocol consists of 90 pairs of electric stimulation at the median nerve and is followed by transcranial magnetic stimulation (TMS) of the M1 contralateral to the stimulated median nerve. The interstimulus interval between the median nerve stimulation and the TMS is a critical factor leading to successful induction of M1 plasticity (Stefan et al., 2000; Müller-Dahlhaus et al., 2010). To induce long-term potentiation (LTP)-like neural activity in M1, the somatosensory afferent from the median nerve stimulation should arrive at the same time with or shortly before the TMS of M1 (Wolters et al., 2003; Ziemann et al., 2004; Byblow et al., 2007; Pötter-Nerger et al., 2009). The somatosensory inputs can be alternative from electric stimulation, passive movement to active movement once the spike-timing dependent principle is followed (Stefan et al., 2000; Thabit et al., 2010; Edwards et al., 2014).

In addition to the ipsilateral somatosensory afferent, the signal from the contralateral M1, probably through the transcallosal pathway, can serve as the conditioning input to induce M1 plasticity (Kobayashi et al., 2003; Koganemaru et al., 2009; Rizzo et al., 2009). It renders the possibility that the sensorimotor activation in the contralateral hemisphere may carry an impact on the induction of M1 plasticity. Nevertheless, the interhemispheric input should promptly arrive in a responsive time window to achieve the induction of M1 plasticity.

This study investigates whether intentional, active movements driven by the contralateral M1 influence the M1 plasticity induced by the conventional PAS protocol. It has been reported that the conventional PAS protocol has a responsive variability (Lahr et al., 2016; Minkova et al., 2019). Since the interval between the median nerve stimulation and the TMS is fixed to 25 milliseconds (ms) in the conventional PAS, one of the possible factors causing individual variability would be dispersed conditioning stimuli at M1 corresponding to the individual timing of the somatosensory afferent. If an additional somatosensory input based on the individual reaction time can be activated, the conjoint conditioning stimuli at M1 may be enhanced and able to decrease the uncertainty of the PAS effect. Therefore, the hypothesis of the current study is that the simultaneous arrival of the sensorimotor afferents from both hemispheres may lead to a convergent influence and enhance the PAS effect on the target M1.

## Materials and Methods

### Subjects

In total, 24 right-handed (Oldfield, 1971) healthy subjects (mean age 24.3 ± 2.9 years, 11 women) participated in this study after giving their written informed consent. They all underwent a motor reaction time measurement (see “Measurement of the Mean Reaction Time to the Auditory Stimulation” section), as well as the PAS protocols including two different motor reacting conditions, respectively (see “Paired Associated Stimulation” and “Paired Associated Stimulation With Simultaneous Motor Reaction at the Contralateral Hand” section). The experimental procedures were in accord with the latest revision of the Declaration of Helsinki. Approval by the local ethics committee of the China Medical University Hospital was obtained (CMUH106-REC2-019).

### Procedures

#### Measurement of Motor Evoked Potentials

TMS was delivered through a focal “figure-of-eight”-shaped stimulating coil (with 70 mm inner diameter of each ring) connected *via* a BiStim moiety to two Magstim 200 magnetic stimulators (Magstim Co., Carmarthenshire, Wales, UK). The optimal coil position (“hot spot”; M1_HAND_) was determined as the site on the left primary motor cortex where the TMS at a supra-threshold intensity consistently produced the largest MEPs in the right APB. The test intensity of the TMS, which was adjusted to produce MEPs around 1 mV in peak-to-peak amplitude on average in the resting APB, is defined as the MEP_1 mV_. The individual resting motor threshold (RMT) and active motor threshold (AMT) of each subject was respectively determined over the left M1_HAND_. The method we applied for individual RMT and AMT determination has been described in the previous literature (Lu et al., 2012). In the measurement of MEPs, 20 stimuli were recorded at the test intensity level. The inter-stimulus interval (ISI) was determined as 10 s with a 25% variance to limit the anticipation effect.

**Figure 1:**
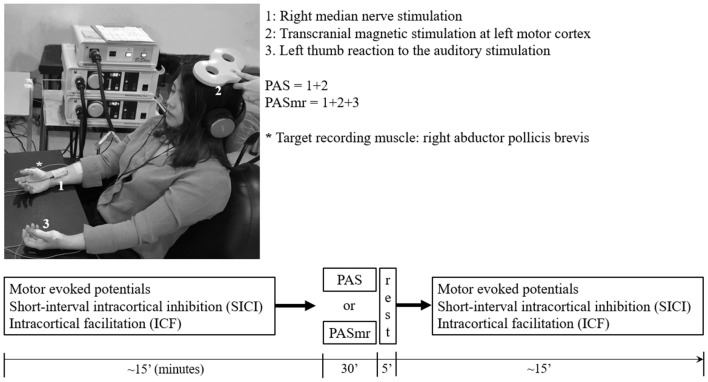
The experimental setup demonstrated by the authors. The subject sat on a chair with his or her arms relaxed and in a supination position. In the “paired associative stimulation (PAS)” protocol, 90 pairs of the transcranial magnetic stimulation (TMS) was delivered to the left motor cortex, 25 ms following the electric stimulation at the right wrist (1 + 2). The motor response was monitored by the surface electromyography at bilateral abductor pollicis brevis (APB) muscles. The earphone was used to receive the sound command triggered from the software in the computer. In the “paired associated stimulation with simultaneous motor reaction at the contralateral hand (PASmr)” protocol, the subjects were requested to move their left APB muscle as quickly as they could (1 + 2 + 3). The experimental design and time flow are shown in the lower panel. All subjects completed the PAS and the PASmr protocols with an interval of at least 1 week.

#### Short-Interval Intracortical Inhibition (SICI)/Intracortical Facilitation (ICF)

Short-interval intracortical inhibition (SICI) and intracortical facilitation (ICF) were studied with the application of the paired-pulse TMS protocol, which had been well-established in the previous literature (Kujirai et al., 1993; Ziemann et al., 1996). In brief, through using the same “figure-of-eight” stimulating coil over the left M1_HAND_, the two magnetic stimuli were given in order to investigate the effect of the sub-threshold conditioning stimulus (Antal et al., 2000) on the test motor evoked potential (MEP) provoked by the supra-threshold test stimulus (TS). SICI was assessed at an ISI of 2.0 ms owing to the results of previous literature that the SICI at this level is not interfered by short-interval intra-ortical facilitation (SICF; Peurala et al., 2008). At the baseline recording, the CS intensity was adjusted to produce approximately 50% inhibition in order to provide the highest sensitivity for the detection of changes in SICI after PAS. AMT had been determined over the left M1 prior to the baseline recording, and 95% AMT was set as the CS intensity, which was kept constant throughout the SICI and ICF measurements. In total twelve single and twelve paired TMS were delivered with a pseudorandomized order to measure the SICI and ICF. The ISI was 10 s with a 25% variance.

#### Measurement of the Mean Reaction Time to the Auditory Stimulation

The reaction time of all subjects was measured by the following equipment and process. Through a pair of earphones connected to the speaker (MDR-XD150, Sony Taiwan Limited) digitally triggered by a computer-based interface (Signal for Windows, Version 3.10, CED, UK), a digital sound with the volume set as 64 dB, the duration as 50 ms, and the frequency as 100 Hz, was transmitted to the subject. The sound was repeated 10 times with an interval of 5 ± 25% seconds (i.e., 3.75–6.25 s). When hearing the sound through the abovementioned settings, every subject was requested to perform the contraction of the left abductor pollicis brevis (APB) muscle as quickly as possible. Surface electromyography (SEMG) was recorded from the left APB muscle to measure each “motor reaction time” span, which was calculated as the period from the starting time of the sound to the onset of the APB muscle activity. The mean reaction time was obtained by averaging ten total measurements of the single reaction time value for each subject.

#### Paired Associated Stimulation (PAS)

The PAS protocol consists of 90 pairs of a cutaneous electric stimulation of the median nerve at the right wrist followed by a single TMS at the contralateral M1_HAND_ (Stefan et al., 2000). Every single electric stimulation over the median nerve was set 45 ms after an occurrence of a digital sound with the volume set as 64 dB, duration as 50 ms, and frequency as 100 Hz in control, with all subjects keeping their left abductor pollicis brevis (APB) in a relaxed condition through the protocol. The interstimulus interval between the electric stimulation of the median nerve and the TMS is 25 ms, as the method of PAS originally been applied in the pioneered study (Stefan et al., 2000). The intensity of TMS was the MEP_1 mV_. In total 90 pairs of PAS were delivered at a rate of 0.05 Hz (i.e., duration of 30 min, Figure 1).

#### Paired Associated Stimulation With Simultaneous Motor Reaction at the Contralateral Hand (PASmr)

The PAS with simultaneous motor reaction (PASmr) protocol consists of 90 “triads” of stimuli, which were each composed of: an instant volitional left APB motor reaction to an auditory stimulation, a cutaneous electric stimulation of the median nerve at the right wrist, and a single TMS at the contralateral M1. Throughout the PASmr protocol, a digital sound with the volume set as 64 dB, the duration as 50 ms, and the frequency as 100 Hz was transmitted through a pair of earphones connected to the speaker (MDR-XD150, Sony Taiwan Ltd.), digitally triggered by a computer-based interface (Signal for Windows, Version 3.10, CED, UK). In the meanwhile, 45 ms after every auditory stimulation of digital sound, which our subject should instantly react to and perform the left APB muscle contraction as quickly as possible, a pair of PAS (see “Paired Associated Stimulation” section) followed. The intensity of TMS was adjusted to the MEP_1 mV_. In total 90 “triads” of PASmr were delivered at a rate of 0.05 Hz (i.e., duration of 30 min). All participants received the PAS and the PASmr protocols with a pseudorandomized order and with an interval of at least 1 week. The experiment setup and design are demonstrated in Figure 1.

### Statistical Analysis

Repeated measures analysis of variance (rmANOVA) was applied to test the intervention effects on the MEP, SICI, and ICF. The within-subject effects were time (pre vs. post) and protocol (PAS vs. PASmr). Conditional on a significant *F*-value, *post hoc* comparisons were performed using paired-sample *t*-tests with Bonferroni’s correction. Violation of sphericity was checked with Mauchly’s test and degrees of freedom were adjusted whenever Mauchly’s *W* < 0.05 using the Greenhouse-Geisser correction (SPSS 22.0). The relationship between reaction time and change of MEP, SICI, and ICF following PAS and PASmr was examined with simple linear regression. Data are reported as means ± SD if not stated otherwise.

## Results

All the 24 subjects completed the whole sessions of experimental procedures without any adverse effects during or after the study.

The mean RMT and AMT of the 24 participants were 53 ± 6.9% and 42 ± 5.8%, respectively. The mean MEP_1 mv_ was 59 ± 8.2%. The intensities applied for measuring SICI and ICF are listed in Table 1. We analyzed the data of the 24 subjects for two main effects (i.e., protocol and time). RmANOVA of the MEP amplitude revealed a significant effect of time (*F*_(1,23)_ = 6.21, *P* = 0.02; Table 2). A significant effect of time was also found for the analysis of SICI (*F*_(1,23)_ = 6.09, *P* = 0.02). RmANOVA of the ICF did not show any significant effect (all *P* > 0.08). The statistical power reached 0.96 with the effect size of 0.4 for the two-way rmANOVA.

**Table 1:** Transcranial magnetic stimulation (TMS) stimulation parameters.

	MEP	SICI	ICF
Conditioning intensity^*^		40 ± 5	40 ± 5
Test intensity^*^	59 ± 8	59 ± 8	59 ± 8

*Presented as percentage of maximal stimulator output.

**Table 2:** Repeated measures analysis of variance (rmANOVA) of the paired associated stimulation with simultaneous motor reaction at the contralateral hand effect.

		MEP		SICI		ICF	
	*d.f*.	*F*	*P*	*F*	*P*	*F*	*P*
Protocol^a^	1.23	1.966	0.174	0.259	0.616	0.031	0.863
Time^b^	1.23	6.210^*^	0.020^*^	6.090^*^	0.021^*^	1.087	0.308
Protocol X Time	1.23	1.850	0.187	1.314	0.263	1.006	0.326

*P < 0.05. ^a^2 levels (PAS and PASmr). ^b^2 levels (pre and post). Abbreviations: *d.f*., degrees of freedom; ICF, intracortical facilitation; MEP, motor evoked potential; SICI, short-interval intracortical inhibition. The bold values mean statistical significance.

**Figure 2:**
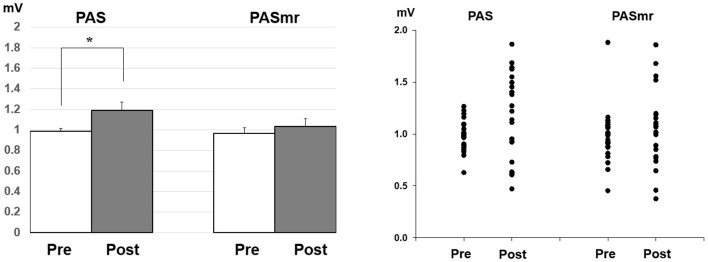
*Post hoc* comparisons of the motor evoked potential (MEP) and the data plots. The MEP showed a significant facilitation following the PAS protocol instead of the PASmr protocol (*P < 0.05 by paired *t*-test with Bonferroni’s correction).

**Figure 3:**
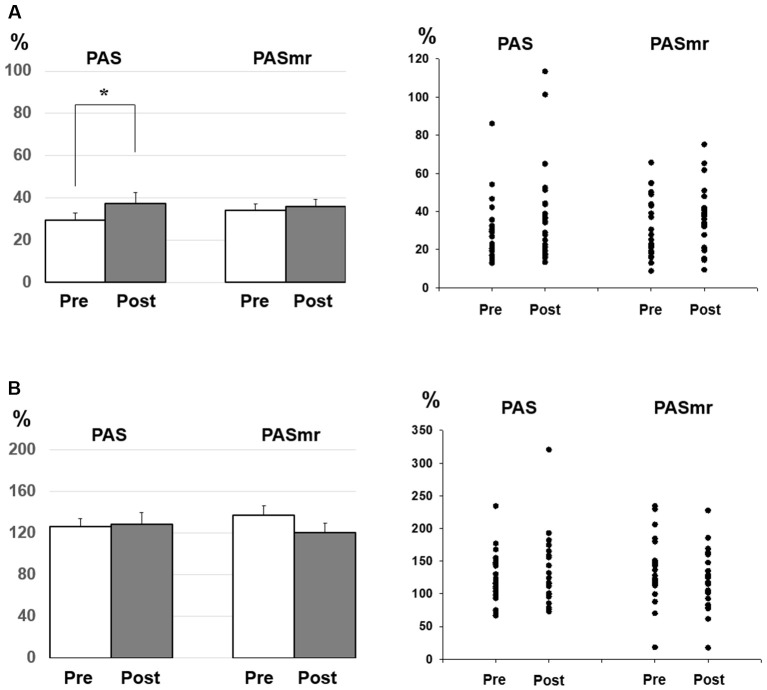
**(A)** *Post hoc* comparisons of the short-interval intracortical inhibition (SICI) and the data plots. The SICI showed a significant reduction following the PAS protocol but not the PASmr protocol (*P < 0.05 by paired *t*-test with Bonferroni’s correction). **(B)** *Post hoc* comparisons of the intracortical facilitation (ICF) and the data plots. There was no significant change following the PAS and the PASmr protocol.

**Figure 4:**
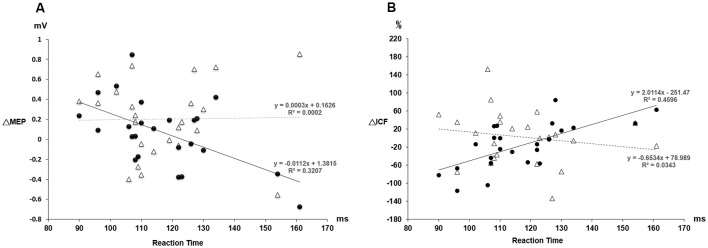
**(A)** The relationship between the MEP change and the individual reaction time. The triangle values with the dot line were measured from the PAS protocol and the black dot values with the solid line were obtained from the PASmr protocol. There shows a significant negative correlation between the MEP change and the reaction time in the PASmr protocol (*R*^2^ = 0.32, *P* = 0.004). **(B)** The relationship between the ICF change and the individual reaction time. There is a significant positive correlation between the ICF change and the reaction time in the PASmr protocol (*R*^2^ = 0.46, *P* < 0.001).

*Post hoc* comparisons of MEP showed a significant MEP amplitude increase after PAS (pre/post: 0.99 ± 0.15/1.19 ± 0.40 mV, *P* = 0.016 by paired *t*-test) but not PASmr (Figure 2). The SICI also showed a significant decrease after PAS (pre/post: 29.6 ± 15.8/37.3 ± 25.5%, *P* = 0.045 by paired *t*-test) but not PASmr (Figure 3A). There was no significant difference from the *post hoc* comparisons on ICF (Figure 3B). The simple linear regression test showed a significant negative correlation between the reaction time and the MEP change in PASmr (*R*^2^ = 0.32, *P* = 0.004) but not PAS (Figure 4A). There was a positive correlation between the reaction time and the change of ICF in PASmr (*R*^2^ = 0.46, *P* < 0.001) but not PAS (Figure 4B). Considering the two subjects with a long reaction time, as this might interfere with the current correlation findings, we re-analyzed the correlations without the two subjects. The correlation between the reaction time and the MEP change did not reach a statistical significance despite a weak trend remaining (*R*^2^ = 0.09, *P* = 0.18). The positive correlation between the reaction time and the change of ICF in PASmr was not affected (*R*^2^ = 0.39, *P* = 0.0019).

**Figure 5:**
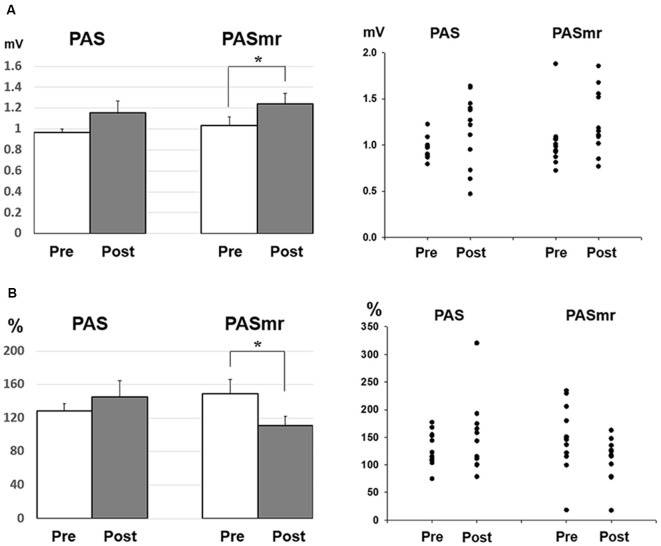
**(A)** *Post hoc* comparisons of the MEP obtained from the subjects with a reaction time of less than 110 ms (*N* = 12) and the data plots. The MEP shows a significant facilitation following the PASmr protocol but not the PAS protocol (*P* = 0.035 by paired *t*-test with Bonferroni’s correction). **(B)** *Post hoc* comparisons of the ICF obtained from the subjects with a reaction time of less than 110 ms (*N* = 12) and the data plots. The ICF shows a significant reduction following the PASmr protocol but not the PAS protocol (*P* = 0.021 by paired *t*-test with Bonferroni’s correction *P < 0.05).

**Figure 6:**
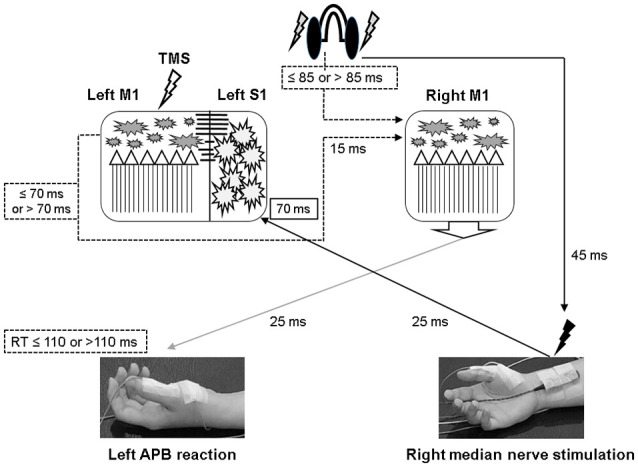
Illustration of the proposed timing flow in bilateral M1. The solid line represents the accurate time period and the dot line indicates the assumed time period. In the PASmr protocol, the somatosensory afferent coming from the right median nerve stimulation arrives at the left somatosensory cortex (S1) approximately 70 ms after the digital sound (shown by the earphones). During the same period, the subjects have to move their left abductor pollicis brevis (APB) muscle to respond to the sound. Since the latency of right M1 to left APB is maximally around 25 ms, a reaction time of 110 ms indicates that the right M1 has to activate corticospinal neurons in 85 ms following the digital sound (that is, 110–25 = 85). Considering that the left M1 needs to disinhibit right M1 through the transcallosal pathway which takes around 15 ms, the left M1 in those subjects with a reaction time of less than 110 ms is supposed to be activated about 70 ms or less following the digital sound (85–15 = 70). Therefore, convergent sensorimotor commands occur almost at the same time when TMS is delivered to the left M1, which takes place in 70 ms following the digital sound.

Since there were certain relationships between the reaction time and the measured electrophysiological parameters, we compared the MEP, SICI, and ICF by classifying the results of all 24 subjects into two groups, based on their motor reaction time (see “Measurement of the Mean Reaction Time to the Auditory Stimulation” section), with one group having an average reaction time of less than 110 ms and the other group more than 110 ms. There were twelve subjects in each group. In the group with a reaction time of more than 110 ms, there was no significant change of MEP, SICI, and ICF following the PAS and PASmr. Nevertheless, in the group with a reaction time of less than 110 ms, the comparisons on MEP amplitude showed a significant increase in PASmr (pre/post: 1.03 ± 0.29/1.24 ± 0.34 mV, *P* = 0.035 by paired *t*-test, Figure 5A). The ICF showed a significant reduction in PASmr (pre/post: 148.9 ± 60.0/111.1 ± 38.5%, *P* = 0.021 by paired *t*-test, Figure 5B). The timing relationship among sensorimotor cortex activation, PAS, and reaction time in this study is demonstrated in Figure 6.

## Discussion

PAS can facilitate MEP with the interstimulus interval of 25 ms between the electrical median nerve stimulation and the TMS of M1, which follows the spike-timing dependent principle (Stefan et al., 2000). In addition to electrical stimulation, intentional hand or foot movement paired with TMS in a specific time interval can alter M1 excitability as well, probably through another somatosensory afferent (Koeneke et al., 2006; Thabit et al., 2010; Mrachacz-Kersting et al., 2012). In principle, the input sensory information should simultaneously or precedingly arrive at M1 while the paired supra-threshold TMS is delivered so the STDP can be generated in M1. In case the sensory afferent is accomplished through complex pathways such as electroacupuncture, the long-term plasticity-like effect cannot be observed (Huang et al., 2019). The current findings support the notion that the spike-timing dependent principle remains following conditions with multiple sensory afferents if the individual difference of the sensory afferents is carefully considered. In this study, the LTP-like MEP facilitation was found in the PAS protocol but not in the PASmr protocol when all subjects were grouped and analyzed together. Since the reaction time played an important role in the arrival of the second sensory input to M1, the grouped analysis with heterogeneous reaction time may average out and conceal the real response (see Figure 2). The fact that the MEP change was significantly correlated with the individual reaction time in the PASmr protocol but not in the PAS protocol suggests the importance of the reaction time on the measured aftereffects in a group level, although our interpretation is inevitably constrained by the limited number of subjects including two cases with a significantly long reaction time (see Figures 4A, 5A).

The MEP was significantly facilitated after the PASmr protocol in whose reaction time was less than 110 ms. For the subjects with a reaction time of 110 ms or less, their right M1 needs to be activated less than 85 ms after the sound was heard (i.e., 110 ms minus 25 ms, a maximal latency of somatosensory input from left APB muscle). At the same time, the left M1 needs to disinhibit right M1 by forwarding a disinhibition signal, probably through the transcallosal pathway mediating interhemispheric inhibition (Ferbert et al., 1992; Meyer et al., 1995, 1998; Kobayashi et al., 2003; Ni et al., 2009). It will take around 10–15 ms for a signal transmitting through the corpus callosum (Brown et al., 1991; Meyer et al., 1995). Therefore, the left M1 is activated about 70 ms after the sound was heard, 15 ms prior to the right M1 activation. The 15 ms interval is the same as the interval reported to be able to induce LTP-like phenomenon with paired TMS at bilateral M1 in humans (Koganemaru et al., 2009). In the left M1, the somatosensory input from the right median nerve electric stimulation and the transcallosal signal for disinhibiting right M1 are convergent almost at the same time in those subjects with a reaction time of less or equal to 110 ms (see Figure 6). The findings suggest that multiple convergent sensory inputs can induce long-term plasticity-like effects if the spike-timing dependent principle for each sensory input is fit.

The SICI was reduced in the PAS protocol (see Figure 3A). It has been reported that the excitability-enhancing PAS protocol (i.e., PAS with an interstimulus interval of 25 ms) may reduce SICI in the condition of a high baseline SICI (Russmann et al., 2009; Lu et al., 2016, 2017). In the current study the baseline SICI reached 29.6 ± 15.8%, it would be ranged within a high level of SICI. It is intriguing that the SICI findings were inconsistent between the PAS protocol and the PASmr protocol. There is no significant difference of SICI in the PASmr protocol. In addition to the somatosensory afferent, the transcallosal afferent may be engaged in the PASmr protocol. Previous studies have shown that interhemispheric inhibition (Hatta et al., 2003) interferes with SICI and long-latency afferent inhibition in the target hemisphere (Kukaswadia et al., 2005; Reis et al., 2008). It is possible that a complex interaction between the IHI, SICI, and corticospinal pathways erases the SICI reduction observed in the PAS protocol.

ICF is thought to be mediated by distinct mechanisms from SICI and corticospinal excitability (Chen et al., 1998; Pyndt and Ridding, 2004). The fact that the change of ICF is significantly correlated with the reaction time in the PASmr protocol indicates that ICF is associated with the motor task performed ipsilateral to the tested M1 (see Figures 3B, 4B, 5B). The previous study has shown that not only the MEP but also the ICF was significantly enhanced during the time the subjects were executing a simple motor task with direct and mirror visual feedback at the same side (Maeda et al., 2002; Garry et al., 2005; Nojima et al., 2012; Kumru et al., 2016). The discrepancy of the ICF change between the previous and the current study can be attributed to the different time period while measuring ICF, during the motor task in the previous study and after the motor task in this study. In addition, the frequency of the motor task (2–3 Hz vs. 0.05 Hz) may also play a role in mediating distinct ICF change. It is noted that the ICF change was only found at the M1 controlling the specific muscle mirror to the contralateral movement side (Kumru et al., 2016). Since the PAS effect is also reported with a topographic specificity (Stefan et al., 2000; Wolters et al., 2003; Michou et al., 2013), the subjects were requested to move their APB muscle mirror to the measured site in this study. A further study investigating the relationship between ICF and motor response is anticipated.

## Conclusions

The findings suggest that simultaneous somatosensory afferents from the contralateral hemisphere induce STDP in M1. This kind of plasticity can be represented by MEP and ICF. Furthermore, the individual motor reaction time is found significantly correlated with the degree of the plasticity. Findings provide evidence for designing novel rehabilitation programs concerning motor function.

## Data Availability Statement

All datasets presented in this study are included in the article.

## Ethics Statement

The studies involving human participants were reviewed and approved by the local ethics committee of the China Medical University Hospital (CMUH106-REC2-019). The patients/participants provided their written informed consent to participate in this study. Written informed consent was obtained from the individual(s) for the publication of any potentially identifiable images or data included in this article.

## Author Contributions

YH: subject recruitment, acquisition of data, and writing the first draft. J-CC: experimental design, critical review of the manuscript, and revision of the first draft. C-HT: experimental design, critical review of the manuscript, and comments on the manuscript. M-KL: study concept and experimental design, data analysis and interpretation, and critical revision of the manuscript. All authors contributed to the article and approved the submitted version.

## Conflict of Interest

The authors declare that the research was conducted in the absence of any commercial or financial relationships that could be construed as a potential conflict of interest.
